# Assessing Evidence Bias for Prehospital Tourniquet Use: A Scoping Review

**DOI:** 10.1002/wjs.12596

**Published:** 2025-05-14

**Authors:** Linda Johansson, Hannah Wild, Jamieson O'Marr, Yves Aziz Nacanabo, Zakaria A. Zeba, Yves Sanou, Serhii V. Tertyshnyi, Teresa Jewell, Henrik Hedelin

**Affiliations:** ^1^ Faculty of Emergency Medicine Sahlgrenska University Hospital Institute of Clinical Sciences Sahlgrenska Academy Gothenburg University Gothenburg Sweden; ^2^ Department of Surgery University of Washington Seattle Washington USA; ^3^ Explosive Weapons Trauma Care Collective International Blast Injury Research Network University of Southampton Southampton UK; ^4^ Department of Orthopaedic Surgery University of California San Francisco San Francisco California USA; ^5^ Captain Hallassane Coulibaly Military Hospital Ouagadougou Burkina Faso; ^6^ Military Medical Clinical Center of the South Region of the Ministry of Defense of Ukraine Odesa Ukraine; ^7^ Health Sciences Library University of Washington Seattle Washington USA; ^8^ Institute of Clinical Sciences Sahlgrenska Academy University of Gothenburg Gothenburg Sweden; ^9^ Department of Orthopaedics NU‐Hospital Group Trollhättan Sweden

**Keywords:** hemorrhage, low‐income countries (LIC), low‐middle income countries (LMIC), prehospital, tourniquets, trauma

## Abstract

**Background:**

The available evidence for the prehospital use of tourniquets is largely based on the military experience of high‐resource countries over the last 30 years. Though the lifesaving benefits of an appropriately applied tourniquet to stop catastrophic bleeding are undeniable, the potential consequences of tourniquet application differ greatly within different environments. In many low resource settings, both the time until initial treatment and the evacuation time to a qualified medical care can range from hours to days. The global burden of trauma disproportionally affects low‐ and middle‐income countries, however the guidelines and training regimes surrounding tourniquet use are based upon data collected from high resource settings may not be directly transferable to these settings.

**Methods:**

A scoping review was employed to assess the geographical and socioeconomic distribution of the currently available reports with clinical outcomes regarding prehospital tourniquet use. Multiple factors were analyzed including socioeconomic, civilian/military, and geographic variables. Following PRISMA‐ScR guidelines, the search strategy was outlined, and a title/abstract screening followed by a full text screening was performed separately by two qualified physicians. Reports not presenting outcome measures were excluded.

**Results:**

A total of 52 reports involving 27,429 patients were included in this scoping review. Most reports were conducted in the USA (48%) or Iraq/Afghanistan (31%), with all primary authors affiliated with high‐income countries, such as the USA, Israel, and the UK. No reports meeting the inclusion criteria had primary authors based in low‐income countries. The study population was predominantly young (mean age 31 years) and male (89%). Penetrating trauma from ballistic injuries and blasts were the most common mechanisms of injury. The average pre‐hospital tourniquet application time was 146 min (median: 72 min). Mortality was the most frequently reported outcome (85%), followed by limb salvage (63%) and neurological impairment (48%).

**Conclusion:**

This review highlights the limited data on pre‐hospital tourniquet use in resource‐limited settings, creating a gap that risks misinformed interventions in these contexts. The absence of research originating from authors in the low‐income settings being studied is notable. Addressing this disparity is crucial to developing safe and effective guidelines applicable across diverse global healthcare environments.

## Graphical abstract

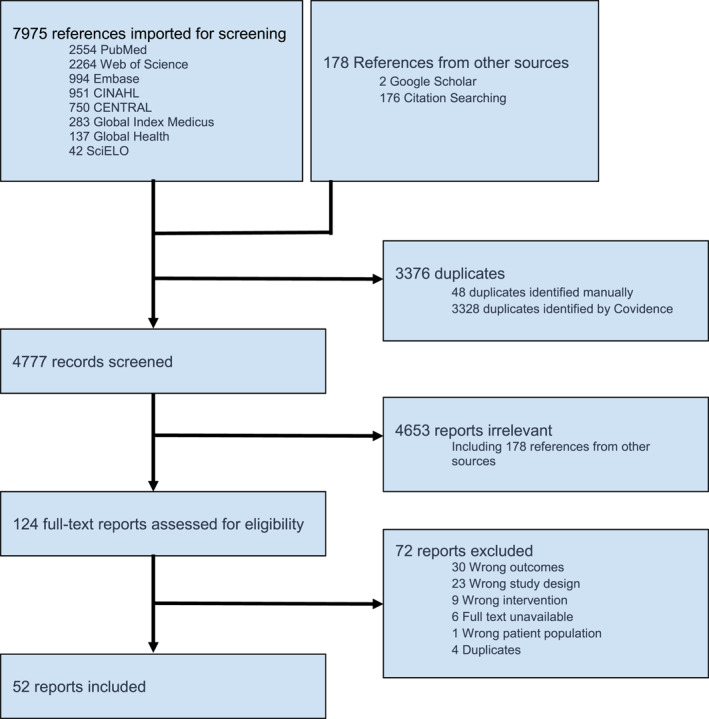

## 1 Introduction

During the last 3 decades, prehospital tourniquet use has been emphasized as a primary mode of hemorrhage control in both civilian and military settings [1]. Previous reviews of the literature have synthesized the evidence for prehospital tourniquet application [2, 3, 4]. However, the majority of this evidence comes from high‐resource settings with organized trauma systems, including prehospital transport and short evacuation times [3, 5]. Global trauma disproportionately affects low‐ and middle‐income countries (LMICs), where coordinated trauma systems are limited and prehospital transport times are frequently prolonged [6, 7, 8]. The life‐saving effect of prompt and appropriate hemorrhage control in general, and the selective use of tourniquets specifically, is established. However, hemorrhage control algorithms as well as training and implementation strategies may differ between different settings. Little evidence exists on optimal modes of prehospital hemorrhage control in low‐resource settings (LRS) [9]. Compositely, this raises concerns about the applicability of available evidence to many civilian contexts worldwide.

Prolonged prehospital tourniquet time, such as that observed in LRS, leads to increased adverse outcomes [10]. The wars in Ukraine and the Sahel have highlighted the dangers of prolonged tourniquet application, even in a country with a relatively high level of trauma care capability such as Ukraine [11, 12, 13, 14, 15]. The consequences of limb loss associated with inappropriate tourniquet use are even more profound in resource‐limited settings with inadequate access to high‐quality prosthetics. Consequently, significant controversy has existed over the appropriateness of teaching tourniquet application in hemorrhage control applications in LRS [16].

A scoping review of the literature was conducted to evaluate the geographic and socioeconomic distribution of authors and study settings as well as resource availability in existing evidence on prehospital tourniquet application in both civilian and military settings. By conducting this review, we aimed to assess the existence of a bias of evidence toward high‐resource settings with short prehospital transport times. Disparities or bias in the evidence base for prehospital tourniquet application could suggest that guidelines based on such findings may not adequately address the unique challenges of tourniquet use in more resource‐constrained environments.

## 2 Materials and Methods

This scoping review follows the recommendations from the Preferred Reporting Items for Systematic Reviews and Meta‐Analysis extension for Scoping Reviews (PRISMA‐ScR) [17]. The protocol was registered at the Open Science Framework on the second of January 2024 (https://osf.io/kgh5w).

### 2.1 Research Question

This scoping review addressed the following question: ‘What are the geographic and socioeconomic characteristics of authors and study settings in the published literature on prehospital tourniquet use and its clinical outcomes?’

### 2.2 Eligibility Criteria

Reports were deemed eligible for inclusion that met the following inclusion criteria: (1) presented data on patients who sustained extremity trauma that was treated with a tourniquet (improvised or commercial) in the prehospital setting; and (2) presented an outcome measure either in the form of limb salvage, neurovascular complications, functional outcomes/disability status, metabolic derangements, and/or mortality. Date restrictions limited eligible reports to those describing prehospital tourniquet application after 1990, coinciding with the initiation of the US military's Tactical Combat Casualty Care guidelines to ensure the relevance of evidence reviewed to modern settings [18]. Reports were excluded that employed junctional tourniquets; (3) reports with tourniquets exclusively in settings other than the pre‐hospital setting including other hospital settings or operating rooms; (4) reports that did not have available full‐text articles available for review. No reports were excluded on the basis of language to avoid bias. No reports were excluded on the basis of study design, with the exception of case reports or commentaries that did not present primary data or outcome measures and therefore did not meet the above‐stipulated eligibility criteria.

### 2.3 Search Strategy

Relevant reports were identified through an electronic search of PubMed, EMBASE (Elsevier), CINAHL (EBSCO), Global Index Medicus, CABI Global Health (EBSCO), Cochrane Library (Wiley), Web of Science Core Collection (– SCI‐EXPANDED, SSCI, AHCI, ESCI). The search was performed on 23rd of February 2024 (Supplement 1). Google Scholar was searched using structured search terms (e.g., “prehospital,” “tourniquet,” “hemorrhage control”). The references of all included reports were screened for additional reports.

### 2.4 Data Extraction and Assessment

All records identified through the search were imported into Covidence systematic review software (Veritas Health Innovation, 2024, Melbourne, Australia), where duplicates were automatically removed. Titles and abstracts were initially screened for relevance and eligibility independently by two reviewers (JO, LJ). A subsequent full‐text screening was conducted in duplicate by the same reviewers using predefined eligibility criteria, with any conflicts resolved by a third author (HW or HH). A single foreign language article (Hebrew) was translated by a medical professional fluent in the language prior to independent screening by the two reviewers (JO, LJ). All other non‐English language articles were translated by one of the authors prior to screening. Additional articles identified through Google Scholar (searched on 2024‐11‐13) and reference lists of included reports were screened at the abstract and full‐text levels by one reviewer (LJ).

All remaining eligible reports underwent extraction using a standardized extraction template which included relevant study characteristics; number of participants and basic demographics (age, sex), civilian or military setting, mechanisms of injury, language, country of origin of both the report and authors, the socioeconomic status of the authors' countries (converted into Gross National Income), tourniquet type, total tourniquet time, and whether any of the investigated outcome variables included limb salvage, neurovascular complications, functional outcomes/disability status, metabolic derangements, and/or mortality.

### 2.5 Synthesis of Results

Results were reported in accordance with PRISMA‐ScR guidelines for scoping reviews (Supplement 2). Descriptive statistics were conducted for relevant variables where applicable. The distribution of reports and settings were presented both in narrative text and visually in graphs and figures to highlight pertinent findings (Tables 1, 2, 3). Though heterogeneity in study design and data formats was anticipated to limit pooled analysis, where applicable, results were pooled and presented compositely.

**TABLE 1:** Summary of included reports.

Report	Study design	Study setting (country)	Country of first author	Number of patients with TQ* application	Study setting (civilian/military)	Study setting (rural/urban)	Average TQ time (min)	Summary
Civilian								
Ballas 2017	Retrospective cohort study	France	France	4	Civilian	Rural	—	TQ use in shark bite victims, clinical features of 27 shark attack cases on La Réunion Island.
Barnard 2020	Retrospective cohort study	USA	USA	168	Civilian	Mixed	—	Patients treated with PHTQ** between January 2018 to June 2019 by EMS***.
Bedri 2022	Retrospective cohort study	USA	USA	92	Civilian	Rural	—	Journal review of patients arriving at a trauma center serving rural Iowa with PHTQ.
Callaway 2014	Case report	USA	USA	4	Civilian	Mixed	—	Series of case reports where law enforcement has applied commercial TQs before EMS has arrived on the scene.
Callaway 2017	Case report	USA	USA	1	Civilian	Urban	65	Child in lawn mower accident.
Eikermann 2014	Case report	USA	USA	1	Civilian	Urban	—	Traumatic injuries after the Boston Marathon.
Gallo 2021	Retrospective chart review	USA	USA	535	Civilian	Mixed	—	Patients from a level 1 trauma center who underwent vascular surgery.
Gushing 2023	Retrospective cohort study	USA	USA	211	Civilian	Mixed	62	Dataset composed of patients with PHTQ collected over three and a half years.
Hashima 2019	Prospective observational study	USA	USA	7161	Civilian	Mixed	—	Describing the characteristics and outcomes following prehospital tourniquet use by EMS in the US.
Henry 2021	Retrospective cohort study	USA	USA	944	Civilian	Urban	—	Torniquet use in LA county 2015‐2019 primarily looking at in‐hospital mortality.
Inaba 2015	Retrospective cohort study	USA	USA	87	Civilian	Urban	103	Adult civilians with extremity injury requiring TQ, primarily looking at rate of limb loss.
Kue 2015	Retrospective journal review	USA	USA	98	Civilian	Mixed	15	Descriptive analysis of EMS experience in Boston between 2005 and 2012.
Legare 2022	Retrospective cohort study	USA	USA	622	Civilian	Urban	51	Study included patients who did not have a vascular injury that then had a tourniquet applied anyway. Primary focus were complications.
Leonard 2016	Retrospective cohort study	USA	USA	95	Civilian	Rural	21	Study comparing prehospital TQ, Quikclot or combat gauze in a group of civilians between 2009 and 2014.
Malo 2015	Case report	Canada	Canada	1	Civilian	Rural	> 1020	Gunshot wound in remote village in Canada.
McCarthy 2023	Retrospective cohort study	USA	USA	52	Civilian	Mixed	43	Cases with PHTQ use from 2015 to 2020 in a regional EMS system in the US.
McNickle 2019	Retrospective cohort study	USA	USA	192	Civilian	Urban	78	Comparing patients with and without prehospital TQ placement, the main outcome was blood transfusion within 24 h.
Ode 2015	Retrospective cohort study	USA	USA	56	Civilian	Urban	72	All documented cases of TQ use in one Mecklenburg County 2012 to 2013.
Passos 2014	Retrospective cohort study	Canada	Canada	190	Civilian	Mixed	—	Patients with vascular injuries arriving at trauma centers between 2011 and 2015.
Read 2023	Retrospective cohort study	Australia	Australia	31	Civilian	Mixed	124	Study performed after the implementation of PHTQ in 2016 as a therapeutic option in the Australian guidelines.
Scala 2020	Retrospective cohort study	USA	USA	61	Civilian	Mixed	—	Database extraction from shark incident registry between 2009 and 2019.
Scerbo 2016	Retrospective cohort study	USA	USA	105	Civilian	Mixed	—	Patients arriving at a level‐1 trauma center between October 2008 and May 2013 with a TQ applied by EMS or in the emergency department.
Scerbo 2016	Retrospective cohort study	USA	USA	306	Civilian	Urban	46	All patients arriving at a level 1 trauma center with a TQ in place or that had one placed in the hospital.
Schroll 2015	Retrospective chart review	USA	USA	197	Civilian	Mixed	48	Analysis of patients arriving at nine urban level 1 trauma centers with PHTQ from January 2010 to December 2013.
Schroll 2021	Prospective observational study	USA	USA	1130	Civilian	Mixed	40	Patients with major extremity trauma and prehospital TQ between 2015 and 2020. Only PHTQ were included in the TQ‐group.
Smith 2018	Retrospective cohort study	USA	USA	238	Civilian	Mixed	35	Study of frequency of TQ use and complications over time, adult patients between 2010 and 2018 arriving with extremity injuries at a hospital in New Orleans.
Smith 2021	Retrospective cohort study	Canada	Canada	27	Civilian	Mixed	—	Vascular trauma and its management across Canadian trauma centers.
Taghavi 2021	Prospective case series	USA	USA	2284	Civilian	Mixed	—	Adults with penetrating trauma to the torso and/or proximal extremity, multicenter study with 25 urban trauma centers participating.
Teixeira 2018	Retrospective cohort study	USA	USA	1026	Civilian	Mixed	77	Study examining if TQ use in civilian settings can be compared to studies from military settings.
Thai 2023	Retrospective cohort study	USA	USA	98	Civilian	Urban	—	Evaluation of TQ application and mobility/functional status, study performed at urban civilian trauma center between 2016 and 2021.
Wellme 2021	Retrospective cohort study	Sweden	Sweden	56	Civilian	Mixed	60	Patients admitted to trauma center Karolinska in Stockholm Sweden, with TQ applied. Data collected were indication, complication, duration, definitive injury.
Military								
Beekley 2008	Retrospective cohort study	Iraq	USA	165	Military	Mixed	70	Compared survival rates between TQ and non TQ groups. US military personnel participating in Operation Iraqi Freedom.
Brodie 2007	Retrospective cohort study	Iraq and Afghanistan	UK	107	Military	Mixed	—	Database study with material from registry of UK military personnel in Iraq and Afghanistan.
Clasper 2009	Retrospective cohort study	Iraq and Afghanistan	UK	65	Military	Mixed	60	Continuation and expansion of earlier study, cohort of patients with TQ and fracture. UK military personnel.
Covey 2021	Prospective case series	Iraq	USA	25	Military	Rural	—	Field tourniquets in an austere military environment.
Dunn 2016	Retrospective cohort study	Iraq and Afghanistan	USA	111	Military	Mixed	—	US military personnel with vascular injuries during operation Iraqi Freedom and operation Enduring Freedom.
Dunn 2016	Retrospective cohort study	Iraq and Afghanistan	USA	313	Military	Mixed	—	TQ use in US**** combat soldiers 2003–2011, registry data from Iraq and Afghanistan.
Gale 2022	Retrospective cohort study	Iraq and Afghanistan	USA	3439	Military	Mixed	—	Compared survival and interventions between female and male pediatric casualties, treated by US medical personnel in Iraq/Afghanistan.
Kauvar 2018	Retrospective cohort study	Iraq and Afghanistan	USA	254	Military	Mixed	72	Studying vascular injuries during operation Iraqi Freedom and operation Enduring Freedom 2004–2012.
Kragh 2007	Case report	Afghanistan	USA	1	Military	Urban	960	Case report of a soldier with abnormally long TQ time where limb was preserved.
Kragh 2007	Prospective observational study	Iraq	USA	428	Military	Mixed	78	Outcomes for patients arriving at a military support hospital in Baghdad.
Kragh 2008	Prospective observational study	Iraq	USA	862	Military	Mixed	—	Analysis of collected data in relation to use of TQ in combat casualty care, pooled with data from earlier study (Kragh 2007).
Kragh 2009	Prospective observational study	Iraq	USA	428	Military	Mixed	—	Prospective study of patients with TQ arriving at hospital in Iraq, the majority of patients were young males.
Kragh 2011	Prospective observational study	Iraq	USA	499	Military	Mixed	—	Continuation of previous study at military support hospital in Baghdad (Kragh 2007).
Kragh 2014	Retrospective cohort study	Iraq and Afghanistan	USA	720	Military	Mixed	—	Data from trauma registry of US casualties from military operations in Afghanistan and Iraq, inclusion required transfusion of blood and TQ use.
Kragh 2015	Retrospective cohort study	Iraq and Afghanistan	USA	1272	Military	Mixed	—	Data from injured US military soldiers with TQ use between 2001 and 2010.
Lairet 2011	Prospective observational study	Afghanistan	USA	1003	Military	Rural	—	Mapping prehospital treatments, did not exclusively study patients with TQ.
Lakstein 2002	Retrospective cohort study	Israel	Israel	91	Military	Urban	83	Summary of a series of battlefield tourniquet placements by Israeli Defense Force personnel.
Sabate‐Ferris 2021	Retrospective cohort study	Mali	France	11	Military	Rural	268	Patients with extremity injury and at least 3 h PHTQ.
Schlaifer 2017	Retrospective cohort study	Israel	Israel	90	Military	Mixed	—	Data collected during operation protective edge. Variables included the number of tourniquet applications, caregiver level, tourniquet type etc.
Tsur 2020	Retrospective cohort study	Israel	Israel	1578	Military	Mixed	—	Comparing outcomes before and after CAT***** was implemented as standard in the Israeli special forces.
Wolfin 1999	Case report	Israel	Israel	1	Military	Rural	200	Case report about a soldier in combat that has been injured from ED with injuries requiring TQ use. The patient sustained nerve injuries in all extremities where TQ had been applied.

Abbreviations: CAT, combat application tourniquet; PHTQ, prehospital tourniquet; TQ, Tourniquet; US, United States.

**TABLE 2:** Demographics and TQ statistics of included reports.

Demographics		Reports presenting variable (*N* = 52)	Reports (%)
Sex ‐ male (%)	89.1	37	71.15%
Age ‐ average (years)	31	44	84.62%
TQ statistics			
TQ‐time ‐ average (minutes)	146	27	51.92%
TQ type (%)	—	33	63.46%
– CAT	57.58%	—	—
– Improvized	12.12%	—	—
– Mixed	30.30%	—	—

Abbreviations: CAT, combat application tourniquet; TQ, Tourniquet.

**TABLE 3:** Study statistics, outcomes and mechanisms of injury.

First author	Reports (*N* = 52)	Reports (%)
USA	39	75%
Israel	4	7.69%
Canada	3	5.77%
France	2	3.85%
UK	2	3.85%
Australia	1	1.92%
Sweden	1	1.92%
Study setting (country)		
USA	25	48.08%
Afghanistan/Iraq	16	30.77%
Israel	4	7.69%
Canada	3	5.77%
France	1	1.92%
Australia	1	1.92%
Mali	1	1.92%
Sweden	1	1.92%
Study background		
Civilian	31	59.62%
Military	21	40.38%
Setting		
Rural	9	17.31%
Urban	10	19.23%
Mixed	33	63.46%
Outcomes reported		
Mortality	44	84.62%
Limb salvage	33	63.46%
Neurological impairment	25	48.08%
Metabolical	23	44.23%
Mechanism of injury represented in article		
Penetrating (not specified)	34	65.38%
Blunt (not specified)	15	28.85%
Gun shot wounds	14	26.92%
Explosive	14	26.92%

## 3 Results

### 3.1 Search Results

**FIGURE 1:**
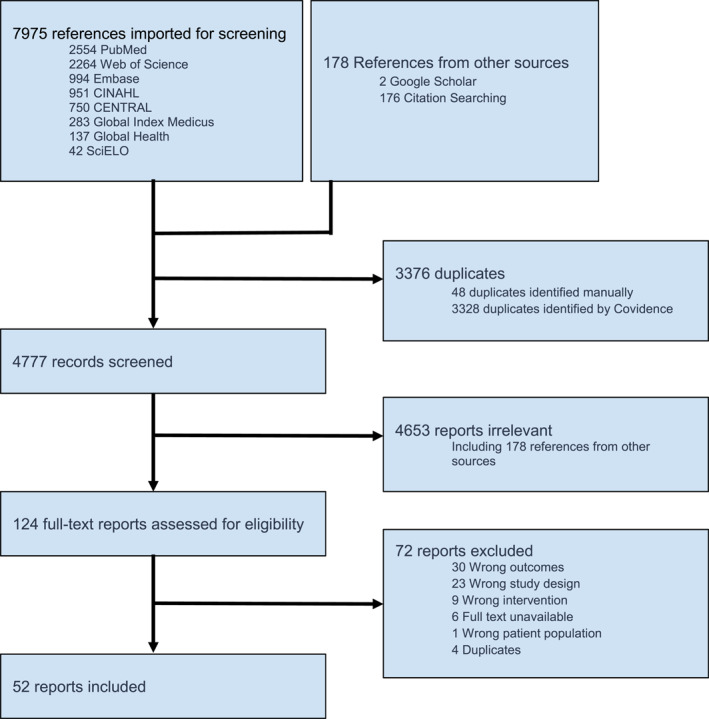
PRISMA‐ScR diagram.

The initial literature search identified 8153 potential references, 3376 of which were identified as duplicates prior to screening and removed. The remaining 4777 reports underwent evaluation with 4653 removed during title and abstract screening and another 72 were removed during full‐text evaluation. Ultimately, 52 reports were included in this scoping review encompassing a total 27,429 patients with pre‐hospital tourniquet application (Table 1). Further study selection information can be found in the PRISMA diagram (Figure 1).

### 3.2 Geographic and Socioeconomic Distribution

**FIGURE 2:**
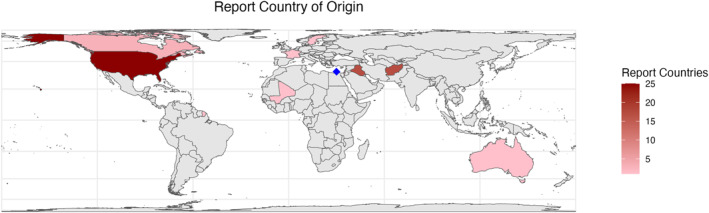
Reports by study setting (country). Blue square marks Israel.

**FIGURE 3:**
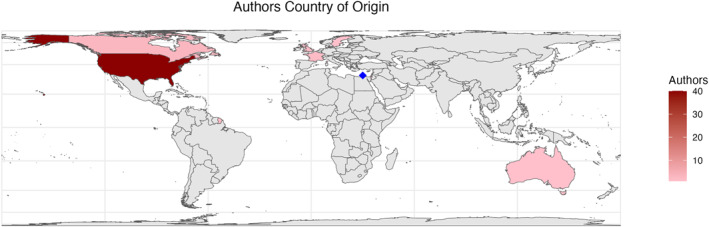
Reports by country of origin of the first author. Blue square marks Israel.

Reports included in analysis presented data from 10 countries. Geographically, reports were conducted in the USA (*n* = 25, 48%), Iraq and Afghanistan (*n* = 16, 31%), Israel (*n* = 4, 8%), Canada (*n* = 3, 6%), France (*n* = 1, 2%), Australia (*n* = 1, 2%), Mali (*n* = 1, 2%) and Sweden (*n* = 1, 2%) (Figure 2). Many authors for the included reports originated from the United States (*n* = 39, 75%). Of the remaining papers, all other authors originated from higher income nations such as Israel, France, the United Kingdom, Canada, Sweden, and Australia. The average gross national income (GNI) per capita of the authors' countries in the included reports was $73,550, compared to $50,600 in the countries where the study was conducted (Figure 3).

### 3.3 Study Design and Setting

This review included six case reports, eight prospective observational reports, and 38 retrospective observational reports. Nine reports (17%) were conducted in rural areas, 10 (19%) in urban areas, and 33 (63%) in mixed settings. Most reports involved civilian populations (*n* = 31, 60%), with the remaining 21 reports (40%) focused on military populations. Patients presented by reports included in analysis were predominantly young (mean age 31 years) and 89% were male (Table 2). Seventeen reports had a study setting classified as LRS but 15 of these were either funded by or performed by the military of a high‐income country (US, UK and France) or the patients received care at a forward medical treatment facility or operating theater belonging to the military. Two of the 17 reports used registry data collected by the military, one of them containing a number of pediatric patients who received care at a military support hospital run by the US Military.

### 3.4 Injury Characteristics, Tourniquet Data, and Patient Outcomes

Penetrating injuries were the most commonly represented mechanism of injury (*n* = 34, 65%), these injuries were predominantly from ballistic injuries and knife wounds. Other mechanisms included motor vehicle crashes (*n* = 5, 10%), shark bites (*n* = 1, 2%) and explosive devices (*n* = 14, 27%). There was a large portion of the studies that only specified blunt trauma as a mechanism of injury (*n* = 15, 29%), as such the different mechanisms of injury included in this pooled group could not be extrapolated and included in any specific subcategories. Only 33 (63%) of the reports reported on the type of tourniquet employed, with 19 (58%) using commercially available tourniquets (CAT), four using improvized tourniquets, and 10 (30%) using a mixture of both. Pre‐hospital tourniquet application time was reported in 27 reports (52%), with an average duration of 146 min and a median of 72 min. Mortality was the most commonly reported outcome (*n* = 44, 85%), followed by limb salvage (*n* = 33,63%), neurological impairment (*n* = 25, 48%), and metabolic outcomes (*n* = 23, 44%) (Table 3).

## 4 Discussion

Key findings of this review include that nearly all eligible reports related to pre‐hospital tourniquet application were produced by high‐income country authors operating in either a high‐resource setting or in a low‐resource setting with significant military and medical resources available. These findings coincided with short total pre‐hospital tourniquet times, with a median of only 72 min in the included reports. In this scoping review, we sought to evaluate the geographic and socioeconomic distribution of authors and study settings in the evidence base for the safety and efficacy of prehospital tourniquet application. Pre‐hospital tourniquet use can be a life‐saving measure when applied correctly to control life‐threatening extremity hemorrhage [19, 20]. However, tourniquets also have the potential to result in serious complications (e.g., compartment syndrome, tissue ischemia requiring amputation) if inappropriately used, which can vary widely based on resource availability, transport speeds and capability, and provider awareness [15].

A minority of the reports presenting outcome data on prehospital tourniquet application had a study population originating in a LRS (*n* = 1, 2%), no first authors of any report included in this review were from LRS. Several reports from LRS‐based authors were identified during initial screening; however, these reports were primarily descriptive or single case reports that did not present clinical outcomes and were therefore not eligible for inclusion [21, 22, 23, 24, 25, 26, 27]. Reports that were conducted in LRS were predominantly conducted by high‐resource authors and studied military personnel from high‐resource nations in the context of a military trauma system [1, 28]. Reports containing mixed military and civilian populations were also performed at a high‐resource military owned hospital [19, 20]. In these reports the majority of patients were still US military personnel and the level of care within the study exceeded the level of resources that is typical for the host nation.

These results can be interpreted to yield two findings: (i) limited high‐quality evidence from LRS exists outside of a military context with intact echelons of care and aeromedical evacuation capabilities to support guidance on prehospital tourniquet use in such environments, and (ii) the gap between the number of reports presenting data from LRS and the absence of first authors from LRS highlights imbalance in representation of LRS authors in academic literature on the topic of interest. Compositely this lack of representation from LRS introduces a bias, limiting the generalizability of current safety and efficacy data regarding prehospital tourniquet use to low‐resource contexts. Existing reports emphasize that LRS often face substantial barriers to prehospital transport, resulting in prolonged transport times to definitive care [29, 30]. Such delays can increase the risks associated with tourniquet use, as toxic metabolites accumulate in the affected limb over time [10]. These complications can be significant, including loss of limb and mortality. Several reports from the ongoing war in Ukraine highlighted the challenges associated with tourniquet use in resource‐limited war zones [12, 13, 14, 29]. In LRS, improperly applied tourniquets can lead to complications rarely encountered in high‐resource settings, emphasizing the importance of proper indication and technique [31, 32, 33, 34, 35, 36, 37].

Data from reports conducted in high‐resource settings, including well‐resourced military trauma systems conducting operations in LRS, cannot reliably inform context‐appropriate hemorrhage control guidelines for trauma care of civilian or host nation military personnel in LRS. To address this gap in the evidence base for prehospital tourniquet application, several measures can be taken. First, more reports are needed presenting primary data from studies conducted in LRS environments, including authors from these settings to ensure guidelines are both applicable and equitable across resource environments. Second, in the absence of such evidence, context‐appropriate training for civilian and military healthcare personnel providing prehospital trauma care in LRS can be adapted and developed based on best practices and available data. Such training must be developed in direct partnership with healthcare personnel from these settings, emphasizing the timing of application of a tourniquet, the pros and cons, as well as the known limitations of this care in LRS. These trainings in turn may emphasize: (i) alternative methods of hemorrhage control (e.g., direct manual pressure, wound packing) and (ii) practical guidance and skills training on appropriate techniques for tourniquet conversion, replacement, and removal.

### 4.1 Limitations

The findings of our study are limited by the quantity and quality of available literature identified through our search methodology. Since eligibility criteria restricted reports included in analysis to those that presented primary data on the outcomes of patients undergoing prehospital tourniquet data, several reports by LRS authors were omitted. However, this approach was taken to demonstrate the disparity in existing evidence in a structured manner. Second, while our search strategy was broad and no search results were excluded on the basis of language, this search strategy relied on indexed literature and search terms developed in English, potentially missing data from non‐English speaking LRS [16]. Third, the outcome measures of the included reports vary greatly regarding for example the presence or absence of associated injuries. Finally, much of the existing research on pre‐hospital tourniquet use consists of case reviews and retrospective reports, which may limit the robustness and generalizability of the evidence described in our review. However, the purpose of this review was not to assess accuracy of data but to conduct a structured assessment of the geographic and socioeconomic distribution of the evidence base for prehospital tourniquet application.

## 5 Conclusion

This scoping review synthesized existing literature on pre‐hospital tourniquet application and outcomes, focusing on the geographic origin of authors and patient populations. Our findings indicate that most current evidence is derived primarily from high‐resource settings and high‐resource military trauma systems in LRS. The lack of data from civilian contexts in LRS highlights a critical gap in understanding the safety and efficacy of prehospital tourniquet use in these settings. This disparity may lead to inaccurate conclusions and poorly informed policy decisions. Addressing this gap by supporting research by LRS authors and developing context‐appropriate training is essential to advancing safe, effective, and evidence‐based hemorrhage control guidelines across diverse healthcare settings.

## Author Contributions

**Linda Johansson:** data curation, formal analysis, investigation, writing – review and editing. **Hannah Wild:** conceptualization, formal analysis, methodology, project administration, supervision, visualization, writing – review and editing. **Jamieson O'Marr:** data curation, formal analysis, writing – review and editing. **Yves Aziz Nacanabo:** resources, writing – review and editing. **Zakaria A. Zeba:** resources, writing – review and editing. **Yves Sanou:** project administration, writing – review and editing. **Serhii V. Tertyshnyi:** resources, supervision, writing – review and editing. **Teresa Jewell:** data curation, methodology, project administration, writing – review and editing. **Henrik Hedelin**: conceptualization, data curation, formal analysis, investigation, methodology, project administration, supervision, validation, visualization, writing – original draft, writing – review and editing.

## Ethics Statement

The authors have nothing to report.

## Conflicts of Interest

The authors declare no conflicts of interest.

## Supporting information

Supporting Information S1

Supporting Information S2
