# Supplementary material for: Assessing Evidence Bias for Prehospital Tourniquet Use: A Scoping Review
Source: World J Surg. 2025 May 14;49(6):1471–83. doi: 10.1002/wjs.12596 (PMC12134191; doi:10.1002/wjs.12596)
Supplement: Supplementary file 1 — Supporting Information S1 [file WJS-49-1471-s001.docx]

**PubMed**

|  | **(Concept: prehospital OR** | **Concept: Injuries)** |
| --- | --- | --- |
| Subject Headings  (MeSH) | "Emergency Medical Services"[Mesh:noexp]  "Advanced Trauma Life Support Care"[Mesh]  "First Aid"[Mesh]  "Emergency Responders"[Mesh]  "Military Medicine"[Mesh] | "Wounds and Injuries"[Mesh:noexp]  "Accidental Injuries"[Mesh]  "Amputation, Traumatic"[Mesh]  "Arm Injuries"[Mesh:noexp]  "Elbow Injuries"[Mesh:noexp]  "Forearm Injuries"[Mesh:noexp]  "Wrist Injuries"[Mesh:noexp]  "Hand Injuries"[Mesh]  "Hip Injuries"[Mesh:noexp]  "Leg Injuries"[Mesh:noexp]  "Ankle Injuries"[Mesh:noexp]  "Foot Injuries"[Mesh:noexp]  "Knee Injuries"[Mesh:noexp]  "Barotrauma"[Mesh:noexp]  "Blast Injuries"[Mesh]  "Bites and Stings"[Mesh:noexp]  "Crush Injuries"[Mesh]  "Fractures, Bone"[Mesh:noexp]  "Ankle Fractures"[Mesh]  "Elbow Fractures"[Mesh]  "Femoral Fractures"[Mesh]  "Fibula Fractures"[Mesh]  "Fracture Dislocation"[Mesh]  "Fractures, Avulsion"[Mesh]  "Fractures, Comminuted"[Mesh]  "Fractures, Compression"[Mesh]  "Fractures, Malunited"[Mesh]  "Fractures, Multiple"[Mesh]  "Fractures, Open"[Mesh]  "Fractures, Stress"[Mesh]  "Humeral Fractures"[Mesh]  "Intra-Articular Fractures"[Mesh]  "Knee Fractures"[Mesh]  "Periprosthetic Fractures"[Mesh]  "Radius Fractures"[Mesh]  "Shoulder Fractures"[Mesh]  "Tibial Fractures"[Mesh]  "Ulna Fractures"[Mesh]  "Wrist Fractures"[Mesh]  "Lacerations"[Mesh]  "Multiple Trauma"[Mesh]  "Occupational Injuries"[Mesh]  "Soft Tissue Injuries"[Mesh]  "Wounds, Penetrating"[Mesh:noexp]  "Wounds, Gunshot"[Mesh]  "Wounds, Stab"[Mesh]  "Hemorrhage"[Mesh:noexp]  "Exsanguination"[Mesh]  "Shock, Hemorrhagic"[Mesh]  **"Accidental Falls"[Mesh]**  **"Accidents, Aviation"[Mesh]**  **"Accidents, Traffic"[Mesh]** |
| Free text terms  (searched in title & abstract [tiab]) | prehospital  pre-hospital  battlefield  "military medicine"  "combat medicine"  "combat care"[tiab:~1]  “military setting*”  “military environment*”  “combat setting*”  “combat environment*”  rural  "remote area*"  "remote setting*"  "remote environment*"  "remote location*"  **austere**  "first responder*"  firefighter*  paramedic*  police  "emergency responder*"  "emergency medical technician*"  EMT  EMTs  "combat medic*"  "rescue personnel"  civilian*  bystander*  layperson*  laypeople  "emergency tourniquet*" | trauma  wound  wounds  injury  injuries  fracture*  laceration*  puncture*  perforat*  "traumatic amputation*"[tiab~1]  hemorrhage  hemorrhages  haemorrhage  haemorrhages  **"bleeding control"[tiab:~1]**  **"bleeding controlling"[tiab:~1]**  **"stopping blood loss"[tiab:~1]**  **"stop the bleed"**  **polytrauma**  **stabbed**  **stabbing**  **stab**  **gunshot***  **gun**  **gunfire**  **firearm***  **bullet***  **knife***  **knives**  **dagger**  **((motor* OR motorbike* OR vehicle* OR road OR traffic OR car OR cars OR cycling OR bicycle* OR automobile* OR bike* OR head on OR pile up) AND (accident* OR crash* OR collision* OR smash*))**  **mvas**  **mva**  **rtas**  **rta** |

|  | **Concept: Tourniquet** |
| --- | --- |
| Subject Headings  (MeSH) | "Tourniquets"[Mesh] |
| Free text terms  (searched in title & abstract [tiab])  or  (searched in [text words](https://pubmed.ncbi.nlm.nih.gov/help/#tw) [tw]) | tourniquet* |

(

"Emergency Medical Services"[Mesh:noexp] OR "Advanced Trauma Life Support Care"[Mesh] OR "First Aid"[Mesh] OR "Emergency Responders"[Mesh] OR "Military Medicine"[Mesh] OR prehospital[tiab] OR pre-hospital[tiab] OR battlefield[tiab] OR "military medicine"[tiab] OR "combat medicine"[tiab] OR "combat care"[tiab:~1] OR “military setting*”[tiab] OR “military environment*”[tiab] OR “combat setting*”[tiab] OR “combat environment*”[tiab] OR rural[tiab] OR "remote area*"[tiab] OR "remote setting*"[tiab] OR "remote environment*"[tiab] OR "remote location*"[tiab] **OR austere[tiab]** OR "first responder*"[tiab] OR firefighter*[tiab] OR paramedic*[tiab] OR police[tiab] OR "emergency responder*"[tiab] OR "emergency medical technician*"[tiab] OR EMT[tiab] OR EMTs[tiab] OR "combat medic*"[tiab] OR "rescue personnel"[tiab] OR civilian*[tiab] OR bystander*[tiab] OR layperson*[tiab] OR laypeople[tiab] OR "emergency tourniquet*"[tiab]

OR

"Wounds and Injuries"[Mesh:noexp] OR "Accidental Injuries"[Mesh] OR "Amputation, Traumatic"[Mesh] OR "Arm Injuries"[Mesh:noexp] OR "Elbow Injuries"[Mesh:noexp] OR "Forearm Injuries"[Mesh:noexp] OR "Wrist Injuries"[Mesh:noexp] OR "Hand Injuries"[Mesh] OR "Hip Injuries"[Mesh:noexp] OR "Leg Injuries"[Mesh:noexp] OR "Ankle Injuries"[Mesh:noexp] OR "Foot Injuries"[Mesh:noexp] OR "Knee Injuries"[Mesh:noexp] OR "Barotrauma"[Mesh:noexp] OR "Blast Injuries"[Mesh] OR "Bites and Stings"[Mesh:noexp] OR "Crush Injuries"[Mesh] OR "Fractures, Bone"[Mesh:noexp] OR "Ankle Fractures"[Mesh] OR "Elbow Fractures"[Mesh] OR "Femoral Fractures"[Mesh] OR "Fibula Fractures"[Mesh] OR "Fracture Dislocation"[Mesh] OR "Fractures, Avulsion"[Mesh] OR "Fractures, Comminuted"[Mesh] OR "Fractures, Compression"[Mesh] OR "Fractures, Malunited"[Mesh] OR "Fractures, Multiple"[Mesh] OR "Fractures, Open"[Mesh] OR "Fractures, Stress"[Mesh] OR "Humeral Fractures"[Mesh] OR "Intra-Articular Fractures"[Mesh] OR "Knee Fractures"[Mesh] OR "Periprosthetic Fractures"[Mesh] OR "Radius Fractures"[Mesh] OR "Shoulder Fractures"[Mesh] OR "Tibial Fractures"[Mesh] OR "Ulna Fractures"[Mesh] OR "Wrist Fractures"[Mesh] OR "Lacerations"[Mesh] OR "Multiple Trauma"[Mesh] OR "Occupational Injuries"[Mesh] OR "Soft Tissue Injuries"[Mesh] OR "Wounds, Penetrating"[Mesh:noexp] OR "Wounds, Gunshot"[Mesh] OR "Wounds, Stab"[Mesh] OR "Hemorrhage"[Mesh:noexp] OR "Exsanguination"[Mesh] OR "Shock, Hemorrhagic"[Mesh] **OR "Accidental Falls"[Mesh] OR "Accidents, Aviation"[Mesh] OR "Accidents, Traffic"[Mesh]** OR trauma[tiab] OR wound[tiab] OR wounds[tiab] OR injury[tiab] OR injuries[tiab] OR fracture*[tiab] OR laceration*[tiab] OR puncture*[tiab] OR perforat*[tiab] OR "traumatic amputation*"[tiab] OR hemorrhage[tiab] OR hemorrhages[tiab] OR haemorrhage[tiab] OR haemorrhages[tiab] **OR "bleeding control"[tiab:~1] OR "bleeding controlling"[tiab:~1] OR "stopping blood loss"[tiab:~1] OR "stop the bleed"[tiab] OR polytrauma[tiab] OR stabbed[tiab] OR stabbing[tiab] OR stab[tiab] OR gunshot*[tiab] OR gun[tiab] OR gunfire[tiab] OR firearm*[tiab] OR bullet*[tiab] OR knife*[tiab] OR knives[tiab] OR dagger[tiab] OR ((motor*[tiab] OR motorbike*[tiab] OR vehicle*[tiab] OR road[tiab] OR traffic[tiab] OR car[tiab] OR cars[tiab] OR cycling[tiab] OR bicycle*[tiab] OR automobile*[tiab] OR bike*[tiab] OR "head on"[tiab] OR "pile up"[tiab]) AND (accident*[tiab] OR crash*[tiab] OR collision*[tiab] OR smash*[tiab])) OR mvas[tiab] OR mva[tiab] OR rtas[tiab] OR rta[tiab]**

)

**AND**

("Tourniquets"[Mesh] OR "tourniquet*"[tiab])

NOT ("Animals"[Mesh] NOT "Humans"[Mesh])

AND (1990:2024[pdat])

2,554 results as of 2/23/24

**Embase; Elsevier**

|  | **(Concept: prehospital OR** | **Concept: Injuries)** |
| --- | --- | --- |
| Subject Headings  (Emtree) | 'emergency health service'/de  'advanced trauma life support'/de  'first aid'/de  'emergency care'/exp  'first responder (person)'/exp  'military medicine'/exp  'combat medic'/exp  'fire fighter'/exp  'police'/de | 'injury'/de  'abrasion'/de  'accidental injury'/exp  'traumatic amputation'/exp  'automutilation'/exp  'avulsion injury'/de  'limb injury'/de  'arm injury'/de  'elbow injury'/de  'forearm injury'/de  'wrist injury'/de  'hand injury'/de  'finger injury'/de  'thumb injury'/de  'leg injury'/de  'ankle injury'/de  'foot injury'/de  'toe injury'/de  'hip injury'/de  'knee injury'/de  'blast injury'/exp  'battle injury'/exp  'bites and stings'/de  'bite'/exp  'dog bite'/exp  'bite wound'/exp  'blood vessel injury'/exp  'crush trauma'/de  'crush fracture'  'bone injury'/de  'fracture'/de  'limb fracture'/exp  'fracture dislocation'/exp  'avulsion fracture'/exp  'comminuted fracture'/exp  'compression fracture'/exp  'greenstick fracture'  'multiple fracture'/exp  'open fracture'/exp  'impaction fracture'/exp  'stress fracture'/exp  'intraarticular fracture'/exp  'joint fracture'/exp  'peri-implant fracture'/exp  'periprosthetic fracture'/exp  'spiral fracture'/exp  'blood vessel perforation'/exp  'laceration'/exp  'multiple trauma'/exp  'occupational accident'/de  'soft tissue injury'/exp  'wound'/de  'gunshot injury'/exp  'knife cut'/exp  'missile wound'/exp  'sharps injury'/exp  'shrapnel injury'/exp  'stab wound'/exp  'penetrating trauma'/de  'bleeding'/de  'exsanguination'/exp  'hemorrhagic shock'/de  'falling'/exp  'transport accident'/exp |
| Free text terms  (searched in title, abstract, and keyword (:ti,ab,kw)) | prehospital  pre-hospital  battlefield  "military medicine"  "combat medicine"  (combat NEAR/2 care)  “military setting*”  “military environment*”  “combat setting*”  “combat environment*”  rural  "remote area*"  "remote setting*"  "remote environment*"  "remote location*"  **austere**  "first responder*"  firefighter*  paramedic*  police  "emergency responder*"  "emergency medical technician*"  EMT  EMTs  "combat medic*"  "rescue personnel"  civilian*  bystander*  layperson*  laypeople  "emergency tourniquet*" | trauma  wound  wounds  injury  injuries  fracture*  laceration*  puncture*  perforat*  (traumatic NEAR/2 amputation*)  hemorrhage  hemorrhages  haemorrhage  haemorrhages  **(bleeding NEAR/2 control*)**  **(stopping NEAR/2 "blood loss")**  **"stop the bleed"**  **polytrauma**  **stabbed**  **stabbing**  **stab**  **gunshot***  **gun**  **gunfire**  **firearm***  **bullet***  **knife***  **knives**  **dagger**  **((motor* OR motorbike* OR vehicle* OR road OR traffic OR car OR cars OR cycling OR bicycle* OR automobile* OR bike* OR "head on" OR "pile up") NEAR/3 (accident* OR crash* OR collision* OR smash*))**  **mvas**  **mva**  **rtas**  **rta** |

|  | **Concept: Tourniquet** |
| --- | --- |
| Subject Headings  (Emtree) | 'tourniquet'/exp |
| Free text terms  (searched in title, abstract, and keyword (:ti,ab,kw)) | tourniquet* |

(

'emergency health service'/de OR 'advanced trauma life support'/de OR 'first aid'/de OR 'emergency care'/exp OR 'first responder (person)'/exp OR 'military medicine'/exp OR 'combat medic'/exp OR 'fire fighter'/exp OR 'police'/de OR (prehospital OR pre-hospital OR battlefield OR "military medicine" OR "combat medicine" OR (combat NEAR/2 care) OR “military setting*” OR “military environment*” OR “combat setting*” OR “combat environment*” OR rural OR "remote area*" OR "remote setting*" OR "remote environment*" OR "remote location*" OR austere OR "first responder*" OR firefighter* OR paramedic* OR police OR "emergency responder*" OR "emergency medical technician*" OR EMT OR EMTs OR "combat medic*" OR "rescue personnel" OR civilian* OR bystander* OR layperson* OR laypeople OR "emergency tourniquet*"):ti,ab,kw

)

**AND**

(

'injury'/de OR 'abrasion'/de OR 'accidental injury'/exp OR 'traumatic amputation'/exp OR 'automutilation'/exp OR 'avulsion injury'/de OR 'limb injury'/de OR 'arm injury'/de OR 'elbow injury'/de OR 'forearm injury'/de OR 'wrist injury'/de OR 'hand injury'/de OR 'finger injury'/de OR 'thumb injury'/de OR 'leg injury'/de OR 'ankle injury'/de OR 'foot injury'/de OR 'toe injury'/de OR 'hip injury'/de OR 'knee injury'/de OR 'blast injury'/exp OR 'battle injury'/exp OR 'bites and stings'/de OR 'bite'/exp OR 'dog bite'/exp OR 'bite wound'/exp OR 'blood vessel injury'/exp OR 'crush trauma'/de OR 'crush fracture' OR 'bone injury'/de OR 'fracture'/de OR 'limb fracture'/exp OR 'fracture dislocation'/exp OR 'avulsion fracture'/exp OR 'comminuted fracture'/exp OR 'compression fracture'/exp OR 'greenstick fracture' OR 'multiple fracture'/exp OR 'open fracture'/exp OR 'impaction fracture'/exp OR 'stress fracture'/exp OR 'intraarticular fracture'/exp OR 'joint fracture'/exp OR 'peri-implant fracture'/exp OR 'periprosthetic fracture'/exp OR 'spiral fracture'/exp OR 'blood vessel perforation'/exp OR 'laceration'/exp OR 'multiple trauma'/exp OR 'occupational accident'/de OR 'soft tissue injury'/exp OR 'wound'/de OR 'gunshot injury'/exp OR 'knife cut'/exp OR 'missile wound'/exp OR 'sharps injury'/exp OR 'shrapnel injury'/exp OR 'stab wound'/exp OR 'penetrating trauma'/de OR 'bleeding'/de OR 'exsanguination'/exp OR 'hemorrhagic shock'/de OR 'falling'/exp OR 'transport accident'/exp OR

(trauma OR wound OR wounds OR injury OR injuries OR fracture* OR laceration* OR puncture* OR perforat* OR (traumatic NEAR/2 amputation*) OR hemorrhage OR hemorrhages OR haemorrhage OR haemorrhages **OR (bleeding NEAR/2 control*) OR (stopping NEAR/2 "blood loss") OR "stop the bleed" OR polytrauma OR stabbed OR stabbing OR stab OR gunshot* OR gun OR gunfire OR firearm* OR bullet* OR knife* OR knives OR dagger OR ((motor* OR motorbike* OR vehicle* OR road OR traffic OR car OR cars OR cycling OR bicycle* OR automobile* OR bike* OR "head on" OR "pile up") NEAR/3 (accident* OR crash* OR collision* OR smash*)) OR mvas OR mva OR rtas OR rta):ti,ab,kw**

)

**AND**

('tourniquet'/exp OR tourniquet*:ti,ab,kw)

NOT ('animal'/exp NOT 'human'/exp)

AND [1990-2024]/py

Limits:

994 results as of 2/23/24

**Cumulative Index to Nursing and Allied Health Literature (CINAHL); EBSCOhost**

|  | **(Concept: prehospital OR** | **Concept: Injuries)** |
| --- | --- | --- |
| Subject Headings (MH) | "Emergency Medical Services"  "Rescue Work+"  "Advanced Trauma Life Support Care"  "First Aid"  "Emergency Responders+"  "Military Medicine" | "Wounds and Injuries"  "Barotrauma"  "Blast Injuries"  "Contusions and Abrasions"  "Accidental Injuries"  "Amputation, Traumatic"  "Arm Injuries"  "Elbow Injuries"  "Forearm Injuries"  "Hand Injuries"  "Shoulder Injuries"  "Leg Injuries"  "Bites and Stings"  "Dog and Cat Bites"  "Bites, Human"  "Crush Injuries"  "Ankle Fractures"  "Avulsion Fractures"  "Elbow Fractures+"  "Femoral Fractures+"  "Fibula Fractures"  "Foot Fractures+"  "Fracture Dislocation"  "Fractures, Comminuted"  "Fractures, Compression+"  "Fractures, Malunited"  "Fractures, Open"  "Fractures"  "Fractures, Stress+"  "Hand Fractures+"  "Humeral Fractures+"  "Knee Fractures+"  "Periprosthetic Fractures"  "Radius Fractures+"  "Shoulder Fractures+"  "Ulna Fractures+"  "Injuries, Self-Inflicted"  "Occupational-Related Injuries"  "Soft Tissue Injuries+"  "Tears and Lacerations"  "Wounds, Penetrating+"  "Sharps Injuries+"  "Wounds, Gunshot"  "Wounds, Stab"  "Trauma"  "Multiple Trauma"  "Hemorrhage"  "Shock, Hemorrhagic"  "Accidental Falls"  "Accidents, Aviation"  "Accidents, Traffic" |
| Free text terms  (searched in Title & Abstract; keyword not a searchable field) | prehospital  pre-hospital  battlefield  "military medicine"  "combat medicine"  (combat N1 care)  “military setting*”  “military environment*”  “combat setting*”  “combat environment*”  rural  "remote area*"  "remote setting*"  "remote environment*"  "remote location*"  **austere**  "first responder*"  firefighter*  paramedic*  police  "emergency responder*"  "emergency medical technician*"  EMT  EMTs  "combat medic*"  "rescue personnel"  civilian*  bystander*  layperson*  laypeople  "emergency tourniquet*" | trauma  wound  wounds  injury  injuries  fracture*  laceration*  puncture*  perforat*  (traumatic N1 amputation*)  hemorrhage  hemorrhages  haemorrhage  haemorrhages  **(bleeding N1 control*)**  **(stopping N1 "blood loss")**  **"stop the bleed"**  **polytrauma**  **stabbed**  **stabbing**  **stab**  **gunshot***  **gun**  **gunfire**  **firearm***  **bullet***  **knife***  **knives**  **dagger**  **((motor* OR motorbike* OR vehicle* OR road OR traffic OR car OR cars OR cycling OR bicycle* OR automobile* OR bike* OR "head on" OR "pile up") N2 (accident* OR crash* OR collision* OR smash*))**  **mvas**  **mva**  **rtas**  **rta** |

|  | **Concept: Tourniquet** |
| --- | --- |
| Subject Headings (MH) | Tourniquets |
| Free text terms  (searched in Title & Abstract; keyword not a searchable field) | tourniquet* |

**( (** MH("Emergency Medical Services" OR "Rescue Work+" OR "Advanced Trauma Life Support Care" OR "First Aid" OR "Emergency Responders+" OR "Military Medicine") OR

TI(prehospital OR pre-hospital OR battlefield OR "military medicine" OR "combat medicine" OR (combat N1 care) OR "military setting*" OR "military environment*" OR "combat setting*" OR "combat environment*" OR rural OR "remote area*" OR "remote setting*" OR "remote environment*" OR "remote location*" OR **austere OR** "first responder*" OR firefighter* OR paramedic* OR police OR "emergency responder*" OR "emergency medical technician*" OR EMT OR EMTs OR "combat medic*" OR "rescue personnel" OR civilian* OR bystander* OR layperson* OR laypeople OR "emergency tourniquet*") OR

AB(prehospital OR pre-hospital OR battlefield OR "military medicine" OR "combat medicine" OR (combat N1 care) OR "military setting*" OR "military environment*" OR "combat setting*" OR "combat environment*" OR rural OR "remote area*" OR "remote setting*" OR "remote environment*" OR "remote location*" OR **austere OR** "first responder*" OR firefighter* OR paramedic* OR police OR "emergency responder*" OR "emergency medical technician*" OR EMT OR EMTs OR "combat medic*" OR "rescue personnel" OR civilian* OR bystander* OR layperson* OR laypeople OR "emergency tourniquet*") **)**

**OR**

**(** MH("Wounds and Injuries" OR "Barotrauma" OR "Blast Injuries" OR "Contusions and Abrasions" OR "Accidental Injuries" OR "Amputation, Traumatic" OR "Arm Injuries" OR "Elbow Injuries" OR "Forearm Injuries" OR "Hand Injuries" OR "Shoulder Injuries" OR "Leg Injuries" OR "Bites and Stings" OR "Dog and Cat Bites" OR "Bites, Human" OR "Crush Injuries" OR "Ankle Fractures" OR "Avulsion Fractures" OR "Elbow Fractures+" OR "Femoral Fractures+" OR "Fibula Fractures" OR "Foot Fractures+" OR "Fracture Dislocation" OR "Fractures, Comminuted" OR "Fractures, Compression+" OR "Fractures, Malunited" OR "Fractures, Open" OR "Fractures" OR "Fractures, Stress+" OR "Hand Fractures+" OR "Humeral Fractures+" OR "Knee Fractures+" OR "Periprosthetic Fractures" OR "Radius Fractures+" OR "Shoulder Fractures+" OR "Ulna Fractures+" OR "Injuries, Self-Inflicted" OR "Occupational-Related Injuries" OR "Soft Tissue Injuries+" OR "Tears and Lacerations" OR "Wounds, Penetrating+" OR "Sharps Injuries+" OR "Wounds, Gunshot" OR "Wounds, Stab" OR "Trauma" OR "Multiple Trauma" OR "Hemorrhage" OR "Shock, Hemorrhagic" OR "Accidental Falls" OR "Accidents, Aviation" OR "Accidents, Traffic") OR

TI(trauma OR wound OR wounds OR injury OR injuries OR fracture* OR laceration* OR puncture* OR perforat* OR (traumatic N1 amputation*) OR hemorrhage OR hemorrhages OR haemorrhage OR haemorrhages **OR (bleeding N1 control*) OR (stopping N1 "blood loss") OR "stop the bleed" OR polytrauma OR stabbed OR stabbing OR stab OR gunshot* OR gun OR gunfire OR firearm* OR bullet* OR knife* OR knives OR dagger OR ((motor* OR motorbike* OR vehicle* OR road OR traffic OR car OR cars OR cycling OR bicycle* OR automobile* OR bike* OR "head on" OR "pile up") N2 (accident* OR crash* OR collision* OR smash*)) OR mvas OR mva OR rtas OR rta)** OR

AB(trauma OR wound OR wounds OR injury OR injuries OR fracture* OR laceration* OR puncture* OR perforat* OR (traumatic N1 amputation*) OR hemorrhage OR hemorrhages OR haemorrhage OR haemorrhages **OR (bleeding N1 control*) OR (stopping N1 "blood loss") OR "stop the bleed" OR polytrauma OR stabbed OR stabbing OR stab OR gunshot* OR gun OR gunfire OR firearm* OR bullet* OR knife* OR knives OR dagger OR ((motor* OR motorbike* OR vehicle* OR road OR traffic OR car OR cars OR cycling OR bicycle* OR automobile* OR bike* OR "head on" OR "pile up") N2 (accident* OR crash* OR collision* OR smash*)) OR mvas OR mva OR rtas OR rta**) **) )**

**AND**

**(**MH("Tourniquets") OR TI(tourniquet*) OR AB(tourniquet*)**)**

**NOT (**(MH "Vertebrates+") NOT (MH "Human")**)**

**AND** PY 1990-

Limits: Academic Journals

951 results as of 2/23/24

**Global Index Medicus**

|  | **(Concept: prehospital OR** | **Concept: Injuries)** |
| --- | --- | --- |
| Subject Descriptors | "Emergency Medical Services"  "Advanced Trauma Life Support Care"  "First Aid"[Mesh]  M01.526.373*  "Military Medicine" | "Wounds and Injuries"  "Accidental Injuries"  C26.062*  "Arm Injuries"  "Elbow Injuries"  "Forearm Injuries"  "Wrist Injuries"  "Hand Injuries"  "Finger Injuries"  "Hip Injuries"  "Leg Injuries"  "Ankle Injuries"  "Foot Injuries"  "Knee Injuries"  "Barotrauma"  "Blast Injuries"  "Bites and Stings"  "Crush Injuries"  "Fractures, Bone"  "Ankle Fractures"  C26.404.020*  C26.404.061*  "Fibula Fractures"  "Fracture Dislocation"  "Fractures, Avulsion"  "Fractures, Comminuted"  "Fractures, Compression"  "Fractures, Malunited"  "Fractures, Multiple"  "Fractures, Open"  "Fractures, Stress"  "Humeral Fractures"  "Humeral Fractures, Distal"  "Intra-Articular Fractures"  C26.404.525*  "Periprosthetic Fractures"  C26.404.562*  "Shoulder Fractures"  "Tibial Fractures"  C26.404.937*  "Wrist Fractures"  "Lacerations"  "Multiple Trauma"  "Occupational Injuries"  "Soft Tissue Injuries"  "Degloving Injuries"  "Wounds, Penetrating"  "Wounds, Gunshot"  "Wounds, Stab"  "Hemorrhage"  "Exsanguination"  "Shock, Hemorrhagic"  "Accidental Falls"  "Accidents, Aviation"  "Accidents, Traffic" |
| Title, abstract, keywords  (ti:(),ab:(),kw:()) | prehospital  pre-hospital  battlefield  "military medicine"  "combat medicine"  "combat care"  "combat casualty care"  "combat trauma care"  "military setting"  "military settings"  "military environment"  "military environments"  "combat setting"  "combat settings"  "combat environment"  "combat environments"  rural  "remote area"  "remote areas"  "remote setting"  "remote settings"  "remote environment"  "remote environments"  "remote location"  "remote locations"  austere  "first responder"  "first responders"  firefighter*  paramedic*  police  "emergency responder"  "emergency responders"  "emergency medical technician"  "emergency medical technicians"  EMT  EMTs  "combat medic"  "combat medics"  "combat medicine"  "rescue personnel"  civilian*  bystander*  layperson*  laypeople  "emergency tourniquet"  "emergency tourniquets" | trauma  wound  wounds  injury  injuries  fracture*  laceration*  puncture*  perforat*  (traumatic AND amputation*)  hemorrhage  hemorrhages  haemorrhage  haemorrhages  "control bleeding"  "controlling bleeding"  "control of bleeding"  "bleeding control"  "stopping blood loss"  "stopping loss of blood"  "stopping the loss of blood"  "stop the bleed"  polytrauma  stabbed  stabbing  stab  gunshot*  gun  gunfire  firearm*  bullet*  knife*  knives  dagger  ((motor* OR motorbike* OR vehicle* OR road OR traffic OR car OR cars OR cycling OR bicycle* OR automobile* OR bike* OR "head on" OR "pile up") AND (accident* OR crash* OR collision* OR smash*))  mvas  mva  rtas  rta |

|  | **Concept: Tourniquet** |
| --- | --- |
| Subject Descriptors | Tourniquets |
| Title, abstract, keywords  (ti:(),ab:(),kw:()) | tw:(tourniquet*) |

**(** mh:("Emergency Medical Services" OR "Advanced Trauma Life Support Care" OR "First Aid" OR M01.526.373* OR "Military Medicine") OR ti:(prehospital OR "pre-hospital" OR battlefield OR "military medicine" OR "combat medicine" OR "combat care" OR "combat casualty care" OR "combat trauma care" OR "military setting" OR "military settings" OR "military environment" OR "military environments" OR "combat setting" OR "combat settings" OR "combat environment" OR "combat environments" OR rural OR "remote area" OR "remote areas" OR "remote setting" OR "remote settings" OR "remote environment" OR "remote environments" OR "remote location" OR "remote locations" OR austere OR "first responder" OR "first responders" OR firefighter* OR paramedic* OR police OR "emergency responder" OR "emergency responders" OR "emergency medical technician" OR "emergency medical technicians" OR EMT OR EMTs OR "combat medic" OR "combat medics" OR "combat medicine" OR "rescue personnel" OR civilian* OR bystander* OR layperson* OR laypeople OR "emergency tourniquet" OR "emergency tourniquets") OR ab:(prehospital OR "pre-hospital" OR battlefield OR "military medicine" OR "combat medicine" OR "combat care" OR "combat casualty care" OR "combat trauma care" OR "military setting" OR "military settings" OR "military environment" OR "military environments" OR "combat setting" OR "combat settings" OR "combat environment" OR "combat environments" OR rural OR "remote area" OR "remote areas" OR "remote setting" OR "remote settings" OR "remote environment" OR "remote environments" OR "remote location" OR "remote locations" OR austere OR "first responder" OR "first responders" OR firefighter* OR paramedic* OR police OR "emergency responder" OR "emergency responders" OR "emergency medical technician" OR "emergency medical technicians" OR EMT OR EMTs OR "combat medic" OR "combat medics" OR "combat medicine" OR "rescue personnel" OR civilian* OR bystander* OR layperson* OR laypeople OR "emergency tourniquet" OR "emergency tourniquets") OR kw:(prehospital OR "pre-hospital" OR battlefield OR "military medicine" OR "combat medicine" OR "combat care" OR "combat casualty care" OR "combat trauma care" OR "military setting" OR "military settings" OR "military environment" OR "military environments" OR "combat setting" OR "combat settings" OR "combat environment" OR "combat environments" OR rural OR "remote area" OR "remote areas" OR "remote setting" OR "remote settings" OR "remote environment" OR "remote environments" OR "remote location" OR "remote locations" OR austere OR "first responder" OR "first responders" OR firefighter* OR paramedic* OR police OR "emergency responder" OR "emergency responders" OR "emergency medical technician" OR "emergency medical technicians" OR EMT OR EMTs OR "combat medic" OR "combat medics" OR "combat medicine" OR "rescue personnel" OR civilian* OR bystander* OR layperson* OR laypeople OR "emergency tourniquet" OR "emergency tourniquets")

OR

mh:("Wounds and Injuries" OR "Accidental Injuries" OR C26.062* OR "Arm Injuries" OR "Elbow Injuries" OR "Forearm Injuries" OR "Wrist Injuries" OR "Hand Injuries" OR "Finger Injuries" OR "Hip Injuries" OR "Leg Injuries" OR "Ankle Injuries" OR "Foot Injuries" OR "Knee Injuries" OR "Barotrauma" OR "Blast Injuries" OR "Bites and Stings" OR "Crush Injuries" OR "Fractures, Bone" OR "Ankle Fractures" OR C26.404.020* OR C26.404.061* OR "Fibula Fractures" OR "Fracture Dislocation" OR "Fractures, Avulsion" OR "Fractures, Comminuted" OR "Fractures, Compression" OR "Fractures, Malunited" OR "Fractures, Multiple" OR "Fractures, Open" OR "Fractures, Stress" OR "Humeral Fractures" OR "Humeral Fractures, Distal" OR "Intra-Articular Fractures" OR C26.404.525* OR "Periprosthetic Fractures" OR C26.404.562* OR "Shoulder Fractures" OR "Tibial Fractures" OR C26.404.937* OR "Wrist Fractures" OR "Lacerations" OR "Multiple Trauma" OR "Occupational Injuries" OR "Soft Tissue Injuries" OR "Degloving Injuries" OR "Wounds, Penetrating" OR "Wounds, Gunshot" OR "Wounds, Stab" OR "Hemorrhage" OR "Exsanguination" OR "Shock, Hemorrhagic" OR "Accidental Falls" OR "Accidents, Aviation" OR "Accidents, Traffic") OR ti:(trauma OR wound OR wounds OR injury OR injuries OR fracture* OR laceration* OR puncture* OR perforat* OR (traumatic AND amputation*) OR hemorrhage OR hemorrhages OR haemorrhage OR haemorrhages OR "control bleeding" OR "controlling bleeding" OR "control of bleeding" OR "bleeding control" OR "stopping blood loss" OR "stopping loss of blood" OR "stopping the loss of blood" OR "stop the bleed" OR polytrauma OR stabbed OR stabbing OR stab OR gunshot* OR gun OR gunfire OR firearm* OR bullet* OR knife* OR knives OR dagger OR ((motor* OR motorbike* OR vehicle* OR road OR traffic OR car OR cars OR cycling OR bicycle* OR automobile* OR bike* OR "head on" OR "pile up") AND (accident* OR crash* OR collision* OR smash*)) OR mvas OR mva OR rtas OR rta) OR ab:(trauma OR wound OR wounds OR injury OR injuries OR fracture* OR laceration* OR puncture* OR perforat* OR (traumatic AND amputation*) OR hemorrhage OR hemorrhages OR haemorrhage OR haemorrhages OR "control bleeding" OR "controlling bleeding" OR "control of bleeding" OR "bleeding control" OR "stopping blood loss" OR "stopping loss of blood" OR "stopping the loss of blood" OR "stop the bleed" OR polytrauma OR stabbed OR stabbing OR stab OR gunshot* OR gun OR gunfire OR firearm* OR bullet* OR knife* OR knives OR dagger OR ((motor* OR motorbike* OR vehicle* OR road OR traffic OR car OR cars OR cycling OR bicycle* OR automobile* OR bike* OR "head on" OR "pile up") AND (accident* OR crash* OR collision* OR smash*)) OR mvas OR mva OR rtas OR rta) OR kw:(trauma OR wound OR wounds OR injury OR injuries OR fracture* OR laceration* OR puncture* OR perforat* OR (traumatic AND amputation*) OR hemorrhage OR hemorrhages OR haemorrhage OR haemorrhages OR "control bleeding" OR "controlling bleeding" OR "control of bleeding" OR "bleeding control" OR "stopping blood loss" OR "stopping loss of blood" OR "stopping the loss of blood" OR "stop the bleed" OR polytrauma OR stabbed OR stabbing OR stab OR gunshot* OR gun OR gunfire OR firearm* OR bullet* OR knife* OR knives OR dagger OR ((motor* OR motorbike* OR vehicle* OR road OR traffic OR car OR cars OR cycling OR bicycle* OR automobile* OR bike* OR "head on" OR "pile up") AND (accident* OR crash* OR collision* OR smash*)) OR mvas OR mva OR rtas OR rta) **)**

**AND**

(mh:("Tourniquets") OR ti:(tourniquet*) OR ab:(tourniquet*) OR kw:(tourniquet*))

AND NOT ((mh: B01.050*) AND NOT mh:(Humans))

AND (year_cluster:[1990 TO 2024])

283 results as of 2/23/24

**Global Health; EBSCOhost**

|  | **(Concept: prehospital OR** | **Concept: Injuries)** |
| --- | --- | --- |
| Descriptors  DE () | "first aid"  "emergencies"  "wound treatment"  "war"  "military areas" | "trauma"  "wounds"  "amputation"  "bites"  "bone fractures"  "haemorrhage"  "accidents"  "falls"  "traffic accidents"  "industrial accidents" |
| Searched in Title & Abstract | prehospital  pre-hospital  battlefield  "military medicine"  "combat medicine"  (combat N1 care)  “military setting*”  “military environment*”  “combat setting*”  “combat environment*”  rural  "remote area*"  "remote setting*"  "remote environment*"  "remote location*"  **austere**  "first responder*"  firefighter*  paramedic*  police  "emergency responder*"  "emergency medical technician*"  EMT  EMTs  "combat medic*"  "rescue personnel"  civilian*  bystander*  layperson*  laypeople  "emergency tourniquet*" | trauma  wound  wounds  injury  injuries  fracture*  laceration*  puncture*  perforat*  (traumatic N1 amputation*)  hemorrhage  hemorrhages  haemorrhage  haemorrhages  **(bleeding N1 control*)**  **(stopping N1 "blood loss")**  **"stop the bleed"**  **polytrauma**  **stabbed**  **stabbing**  **stab**  **gunshot***  **gun**  **gunfire**  **firearm***  **bullet***  **knife***  **knives**  **dagger**  **((motor* OR motorbike* OR vehicle* OR road OR traffic OR car OR cars OR cycling OR bicycle* OR automobile* OR bike* OR head on OR pile up) N2 (accident* OR crash* OR collision* OR smash*))**  **mvas**  **mva**  **rtas**  **rta** |

|  | **Concept: Tourniquet** |
| --- | --- |
| Descriptors  DE () | - |
| Searched in Title & Abstract | tourniquet* |

**( (** DE("first aid" OR "emergencies" OR "wound treatment" OR "war" OR "military areas"OR "barefoot doctors" OR "fire fighters" OR "health care workers" OR "medical auxiliaries" OR "technicians") OR

TI(prehospital OR pre-hospital OR battlefield OR "military medicine" OR "combat medicine" OR (combat N1 care) OR "military setting*" OR "military environment*" OR "combat setting*" OR "combat environment*" OR rural OR "remote area*" OR "remote setting*" OR "remote environment*" OR "remote location*" OR **austere OR** "first responder*" OR firefighter* OR paramedic* OR police OR "emergency responder*" OR "emergency medical technician*" OR EMT OR EMTs OR "combat medic*" OR "rescue personnel" OR civilian* OR bystander* OR layperson* OR laypeople OR "emergency tourniquet*") OR

AB(prehospital OR pre-hospital OR battlefield OR "military medicine" OR "combat medicine" OR (combat N1 care) OR "military setting*" OR "military environment*" OR "combat setting*" OR "combat environment*" OR rural OR "remote area*" OR "remote setting*" OR "remote environment*" OR "remote location*" OR **austere OR** "first responder*" OR firefighter* OR paramedic* OR police OR "emergency responder*" OR "emergency medical technician*" OR EMT OR EMTs OR "combat medic*" OR "rescue personnel" OR civilian* OR bystander* OR layperson* OR laypeople OR "emergency tourniquet*") **)**

**OR**

**(**DE("trauma" OR "wounds" OR "amputation" OR "bone fractures" OR "haemorrhage" OR "accidents" OR "falls" OR "traffic accidents" OR "industrial accidents") OR

TI(trauma OR wound OR wounds OR injury OR injuries OR fracture* OR laceration* OR puncture* OR perforat* OR (traumatic N1 amputation*) OR hemorrhage OR hemorrhages OR haemorrhage OR haemorrhages **OR (bleeding N1 control*) OR (stopping N1 "blood loss") OR "stop the bleed" OR polytrauma OR stabbed OR stabbing OR stab OR gunshot* OR gun OR gunfire OR firearm* OR bullet* OR knife* OR knives OR dagger OR ((motor* OR motorbike* OR vehicle* OR road OR traffic OR car OR cars OR cycling OR bicycle* OR automobile* OR bike* OR "head on" OR "pile up") N2 (accident* OR crash* OR collision* OR smash*)) OR mvas OR mva OR rtas OR rta)** OR

AB(trauma OR wound OR wounds OR injury OR injuries OR fracture* OR laceration* OR puncture* OR perforat* OR (traumatic N1 amputation*) OR hemorrhage OR hemorrhages OR haemorrhage OR haemorrhages **OR (bleeding N1 control*) OR (stopping N1 "blood loss") OR "stop the bleed" OR polytrauma OR stabbed OR stabbing OR stab OR gunshot* OR gun OR gunfire OR firearm* OR bullet* OR knife* OR knives OR dagger OR ((motor* OR motorbike* OR vehicle* OR road OR traffic OR car OR cars OR cycling OR bicycle* OR automobile* OR bike* OR "head on" OR "pile up") N2 (accident* OR crash* OR collision* OR smash*)) OR mvas OR mva OR rtas OR rta**) **) )**

**AND**

**(**TI(tourniquet*) OR AB(tourniquet*)**)**

NOT (DE( "laboratory animals" OR "laboratory mammals" OR "animal experiments" OR "animal models") NOT DE("man"))

Limits: Publication date 1990-2024; Academic Journals

137 results as of 2/23/24

**Cochrane; Wiley**

|  | **(Concept: prehospital OR** | **Concept: Injuries)** |
| --- | --- | --- |
| Subject Headings  [mh] | [mh ^"Emergency Medical Services"]  [mh "Advanced Trauma Life Support Care"]  [mh "First Aid"]  [mh "Emergency Responders"]  [mh "Military Medicine"] | [mh ^"Wounds and Injuries"]  [mh "Accidental Injuries"]  [mh "Amputation, Traumatic"]  [mh ^"Arm Injuries"]  [mh ^"Elbow Injuries"]  [mh ^"Forearm Injuries"]  [mh ^"Wrist Injuries"]  [mh "Hand Injuries"]  [mh ^"Hip Injuries"]  [mh ^"Leg Injuries"]  [mh ^"Ankle Injuries"]  [mh ^"Foot Injuries"]  [mh ^"Knee Injuries"]  [mh ^"Barotrauma"]  [mh "Blast Injuries"]  [mh ^"Bites and Stings"]  [mh "Crush Injuries"]  [mh ^"Fractures, Bone"]  [mh "Ankle Fractures"]  [mh "Elbow Fractures"]  [mh "Femoral Fractures"]  [mh "Fibula Fractures"]  [mh "Fracture Dislocation"]  [mh "Fractures, Avulsion"]  [mh "Fractures, Comminuted"]  [mh "Fractures, Compression"]  [mh "Fractures, Malunited"]  [mh "Fractures, Multiple"]  [mh "Fractures, Open"]  [mh "Fractures, Stress"]  [mh "Humeral Fractures"]  [mh "Intra-Articular Fractures"]  [mh "Knee Fractures"]  [mh "Periprosthetic Fractures"]  [mh "Radius Fractures"]  [mh "Shoulder Fractures"]  [mh "Tibial Fractures"]  [mh "Ulna Fractures"]  [mh "Wrist Fractures"]  [mh "Lacerations"]  [mh "Multiple Trauma"]  [mh "Occupational Injuries"]  [mh "Soft Tissue Injuries"]  [mh ^"Wounds, Penetrating"]  [mh "Wounds, Gunshot"]  [mh "Wounds, Stab"]  [mh ^"Hemorrhage"]  [mh "Exsanguination"]  [mh "Shock, Hemorrhagic"]  [mh **"Accidental Falls"]**  **[mh "Accidents, Aviation"]**  **[mh "Accidents, Traffic"]** |
| Free text terms  :ti,ab | prehospital  "pre-hospital"  battlefield  "military medicine"  "combat medicine"  (combat NEAR/1 care)  ((military OR combat) NEXT (environment* OR setting*))  rural  (remote NEXT (area* OR setting* OR environment* OR location*))  **austere**  (first NEXT responder*)  firefighter*  paramedic*  police  (emergency NEXT responder*)  ("emergency medical" NEXT technician*)  EMT  EMTs  (combat NEXT medic*)  "rescue personnel"  civilian*  bystander*  layperson*  laypeople  (emergency NEXT tourniquet*) | trauma  wound  wounds  injury  injuries  fracture*  laceration*  puncture*  perforat*  (traumatic NEAR/1 amputation*)  hemorrhage  hemorrhages  haemorrhage  haemorrhages  **(bleeding NEAR/1 control)**  **(bleeding NEAR/1 controlling)**  **(stopping NEAR/1 blood NEAR/1 loss)**  **polytrauma**  **stabbed**  **stabbing**  **stab**  **gunshot***  **gun**  **gunfire**  **firearm***  **bullet***  **knife***  **knives**  **dagger**  **((motor* OR motorbike* OR vehicle* OR road OR traffic OR car OR cars OR cycling OR bicycle* OR automobile* OR bike* OR head on OR pile up) NEAR/2 (accident* OR crash* OR collision* OR smash*))**  **mvas**  **mva**  **rtas**  **rta** |

|  | tourniquet |
| --- | --- |
| Subject Headings  [mh] | [mh "Tourniquets"] |
| Free text terms  :ti,ab | tourniquet* |

Search Name:

Date Run: 23/02/2024 23:37:10

Comment:

| ID | Search | Hits |
| --- | --- | --- |
| ID | Search | Hits |
| #1 | [mh ^"Emergency Medical Services"] OR [mh "Advanced Trauma Life Support Care"] OR [mh "First Aid"] OR [mh "Emergency Responders"] OR [mh "Military Medicine"] | 2200 |
| #2 | ( prehospital OR pre-hospital OR battlefield OR "military medicine" OR "combat medicine" OR (combat NEAR/1 care) OR ((military OR combat) NEXT (environment* OR setting*)) OR rural OR (remote NEXT (area* OR setting* OR environment* OR location*)) OR austere OR (first NEXT responder*) OR firefighter* OR paramedic* OR police OR (emergency NEXT responder*) OR ("emergency medical" NEXT technician*) OR EMT OR EMTs OR (combat NEXT medic*) OR "rescue personnel" OR civilian* OR bystander* OR layperson* OR laypeople OR (emergency NEXT tourniquet*) ):ti,ab | 18324 |
| #3 | #1 OR #2 | 19418 |
| #4 | [mh ^"Wounds and Injuries"] OR [mh "Accidental Injuries"] OR [mh "Amputation, Traumatic"] OR [mh ^"Arm Injuries"] OR [mh ^"Elbow Injuries"] OR [mh ^"Forearm Injuries"] OR [mh ^"Wrist Injuries"] OR [mh "Hand Injuries"] OR [mh ^"Hip Injuries"] OR [mh ^"Leg Injuries"] OR [mh ^"Ankle Injuries"] OR [mh ^"Foot Injuries"] OR [mh ^"Knee Injuries"] OR [mh ^"Barotrauma"] OR [mh "Blast Injuries"] OR [mh ^"Bites and Stings"] OR [mh "Crush Injuries"] OR [mh ^"Fractures, Bone"] OR [mh "Ankle Fractures"] OR [mh "Elbow Fractures"] OR [mh "Femoral Fractures"] OR [mh "Fibula Fractures"] OR [mh "Fracture Dislocation"] OR [mh "Fractures, Avulsion"] OR [mh "Fractures, Comminuted"] OR [mh "Fractures, Compression"] OR [mh "Fractures, Malunited"] OR [mh "Fractures, Multiple"] OR [mh "Fractures, Open"] OR [mh "Fractures, Stress"] OR [mh "Humeral Fractures"] OR [mh "Intra-Articular Fractures"] OR [mh "Knee Fractures"] OR [mh "Periprosthetic Fractures"] OR [mh "Radius Fractures"] OR [mh "Shoulder Fractures"] OR [mh "Tibial Fractures"] OR [mh "Ulna Fractures"] OR [mh "Wrist Fractures"] OR [mh "Lacerations"] OR [mh "Multiple Trauma"] OR [mh "Occupational Injuries"] OR [mh "Soft Tissue Injuries"] OR [mh ^"Wounds, Penetrating"] OR [mh "Wounds, Gunshot"] OR [mh "Wounds, Stab"] OR [mh ^"Hemorrhage"] OR [mh "Exsanguination"] OR [mh "Shock, Hemorrhagic"] OR [mh "Accidental Falls"] OR [mh "Accidents, Aviation"] OR [mh "Accidents, Traffic"] | 23852 |
| #5 | (trauma OR wound OR wounds OR injury OR injuries OR fracture* OR laceration* OR puncture* OR perforat* OR (traumatic NEAR/1 amputation*) OR hemorrhage OR hemorrhages OR haemorrhage OR haemorrhages OR (bleeding NEAR/1 control*) OR (stopping NEAR/1 blood NEAR/1 loss) OR polytrauma OR stabbed OR stabbing OR stab OR gunshot* OR gun OR gunfire OR firearm* OR bullet* OR knife* OR knives OR dagger OR ((motor* OR motorbike* OR vehicle* OR road OR traffic OR car OR cars OR cycling OR bicycle* OR automobile* OR bike* OR "head on" OR "pile up") NEAR/2 (accident* OR crash* OR collision* OR smash*)) OR mvas OR mva OR rtas OR rta):ti,ab | 151465 |
| #6 | #4 OR #5 | 159567 |
| #7 | #3 OR #6 | 176656 |
| #8 | [mh "Tourniquets"] | 695 |
| #9 | (tourniquet*):ti,ab | 2455 |
| #10 | #8 OR #9 | 2553 |
| #11 | #7 AND #10 | 751 |
| #12 | #11 NOT ([mh "Animals"] NOT [mh "Humans"]) with Cochrane Library publication date Between Jan 1990 and Dec 2024 | 750 |
| #13 | #12 in Cochrane Reviews | 5 |
| #14 | #13 in Trials | 745 |

**Web of Science Core Collection – SCI-EXPANDED, SSCI, AHCI, ESCI**

|  | **(Concept: prehospital OR** | **Concept: Injuries)** |
| --- | --- | --- |
| Topic  (searches title, abstract, author keywords, Keywords Plus®) | prehospital  pre-hospital  battlefield  "military medicine"  "combat medicine"  (combat NEAR/1 care)  “military setting*”  “military environment*”  “combat setting*”  “combat environment*”  rural  "remote area*"  "remote setting*"  "remote environment*"  "remote location*"  **austere**  "first responder*"  firefighter*  paramedic*  police  "emergency responder*"  "emergency medical technician*"  EMT  EMTs  "combat medic*"  "rescue personnel"  civilian*  bystander*  layperson*  laypeople  "emergency tourniquet*" | trauma  wound  wounds  injury  injuries  fracture*  laceration*  puncture*  perforat*  (traumatic NEAR/1 amputation*)  hemorrhage  hemorrhages  haemorrhage  haemorrhages  **(bleeding NEAR/1 control*)**  **(stopping NEAR/1 "blood loss")**  **"stop the bleed"**  **polytrauma**  **stabbed**  **stabbing**  **stab**  **gunshot***  **gun**  **gunfire**  **firearm***  **bullet***  **knife***  **knives**  **dagger**  **((motor* OR motorbike* OR vehicle* OR road OR traffic OR car OR cars OR cycling OR bicycle* OR automobile* OR bike* OR head on OR pile up) NEAR/2 (accident* OR crash* OR collision* OR smash*))**  **mvas**  **mva**  **rtas**  **rta** |

|  | **Concept: Tourniquet** |
| --- | --- |
| Topic  (searches title, abstract, author keywords, Keywords Plus®) | tourniquet* |

**(**TS=( prehospital OR pre-hospital OR battlefield OR "military medicine" OR "combat medicine" OR (combat NEAR/1 care) OR "military setting*" OR "military environment*" OR "combat setting*" OR "combat environment*" OR rural OR "remote area*" OR "remote setting*" OR "remote environment*" OR "remote location*" OR **austere OR** "first responder*" OR firefighter* OR paramedic* OR police OR "emergency responder*" OR "emergency medical technician*" OR EMT OR EMTs OR "combat medic*" OR "rescue personnel" OR civilian* OR bystander* OR layperson* OR laypeople OR "emergency tourniquet*")

OR

TS=( trauma OR wound OR wounds OR injury OR injuries OR fracture* OR laceration* OR puncture* OR perforat* OR (traumatic NEAR/1 amputation*) OR hemorrhage OR hemorrhages OR haemorrhage OR haemorrhages **OR (bleeding NEAR/1 control*) OR (stopping NEAR/1 "blood loss") OR "stop the bleed" OR polytrauma OR stabbed OR stabbing OR stab OR gunshot* OR gun OR gunfire OR firearm* OR bullet* OR knife* OR knives OR dagger OR ((motor* OR motorbike* OR vehicle* OR road OR traffic OR car OR cars OR cycling OR bicycle* OR automobile* OR bike* OR head-on OR pile-up) NEAR/2 (accident* OR crash* OR collision* OR smash*)) OR mvas OR mva OR rtas OR rta**) **)**

**AND**

TS=(tourniquet*)

Limits: Timespan: 1990-01-01 to 2024-12-31 (Publication Date)

2684 results as of 2/23/24

Full names of collections in UW's Web of Science subscription. Include these in appendix/supplementary material.:

Science Citation Index Expanded, 1900-present

Social Sciences Citation Index, 1900-present

Arts & Humanities Citation Index, 1975-present

Emerging Sources Citation Index, 2005-present

**SciELO (Clarivate)**

|  | **(Concept: prehospital OR** | **Concept: Injuries)** |
| --- | --- | --- |
| Topic  (searches title, abstract, author keywords, Keywords Plus®) | prehospital  pre-hospital  battlefield  "military medicine"  "combat medicine"  (combat NEAR/1 care)  “military setting*”  “military environment*”  “combat setting*”  “combat environment*”  rural  "remote area*"  "remote setting*"  "remote environment*"  "remote location*"  **austere**  "first responder*"  firefighter*  paramedic*  police  "emergency responder*"  "emergency medical technician*"  EMT  EMTs  "combat medic*"  "rescue personnel"  civilian*  bystander*  layperson*  laypeople  "emergency tourniquet*" | trauma  wound  wounds  injury  injuries  fracture*  laceration*  puncture*  perforat*  (traumatic NEAR/1 amputation*)  hemorrhage  hemorrhages  haemorrhage  haemorrhages  **(bleeding NEAR/1 control*)**  **(stopping NEAR/1 "blood loss")**  **"stop the bleed"**  **polytrauma**  **stabbed**  **stabbing**  **stab**  **gunshot***  **gun**  **gunfire**  **firearm***  **bullet***  **knife***  **knives**  **dagger**  **((motor* OR motorbike* OR vehicle* OR road OR traffic OR car OR cars OR cycling OR bicycle* OR automobile* OR bike* OR head on OR pile up) NEAR/2 (accident* OR crash* OR collision* OR smash*))**  **mvas**  **mva**  **rtas**  **rta** |

|  | **Concept: Tourniquet** |
| --- | --- |
| Topic  (searches title, abstract, author keywords, Keywords Plus®) | tourniquet* |

**(**TS=( prehospital OR pre-hospital OR battlefield OR "military medicine" OR "combat medicine" OR (combat NEAR/1 care) OR "military setting*" OR "military environment*" OR "combat setting*" OR "combat environment*" OR rural OR "remote area*" OR "remote setting*" OR "remote environment*" OR "remote location*" OR **austere OR** "first responder*" OR firefighter* OR paramedic* OR police OR "emergency responder*" OR "emergency medical technician*" OR EMT OR EMTs OR "combat medic*" OR "rescue personnel" OR civilian* OR bystander* OR layperson* OR laypeople OR "emergency tourniquet*")

OR

TS=( trauma OR wound OR wounds OR injury OR injuries OR fracture* OR laceration* OR puncture* OR perforat* OR (traumatic NEAR/1 amputation*) OR hemorrhage OR hemorrhages OR haemorrhage OR haemorrhages OR (bleeding NEAR/1 control*) OR (stopping NEAR/1 "blood loss") OR "stop the bleed" OR polytrauma OR stabbed OR stabbing OR stab OR gunshot* OR gun OR gunfire OR firearm* OR bullet* OR knife* OR knives OR dagger OR ((motor* OR motorbike* OR vehicle* OR road OR traffic OR car OR cars OR cycling OR bicycle* OR automobile* OR bike* OR head-on OR pile-up) NEAR/2 (accident* OR crash* OR collision* OR smash*)) OR mvas OR mva OR rtas OR rta) **)**

**AND**

TS=(tourniquet*)

Limits: Timespan: 1990-01-01 to 2024-12-31 (Publication Date)

42 results as of 2/23/24
